# Financing national non-communicable disease responses

**DOI:** 10.1080/16549716.2017.1326687

**Published:** 2017-06-12

**Authors:** Luke Nelson Allen

**Affiliations:** ^a^ Nuffield Department of Primary Care Health Sciences, University of Oxford, Oxford, UK

**Keywords:** Global health, non-communicable diseases, financing, development, health systems

## Abstract

Non-communicable diseases (NCDs) (also known as socially transmitted diseases) were conspicuously absent from the Millennium Development Goals and seemed to miss out on the ‘golden years’ of health funding despite causing more death and disability than any other disease group worldwide. The share of ‘development assistance for health’ dedicated to NCDs has remained at 1–2% of the total since 2000. This level of funding is insufficient to attain the nine targets in the World Health Organization (WHO) Global Action Plan on NCDs. In 2015 the Sustainable Development Goals – which include the target of reducing premature NCD mortality by a third – were endorsed by 193 countries. Whilst this commitment is welcome, the same text stresses the primacy of domestic financing, which is currently dominated by out-of-pocket payments in low- and middle-income countries (LMICs). This paper presents the findings of the WHO Global Coordination Mechanism on NCDs financing working group. The group was convened to explore NCD financing options with an emphasis on LMICs. The main sources of available finance include taxation, loans, engagement with the private sector, impact investment and innovative financing mechanisms. There is a role for development assistance to increase in the interim as raising additional revenue from these sources will take time. In the medium term it may be appropriate for international NCD funding to remain low where LMICs successfully assume financial responsibility for preventing and controlling NCDs. Countries will have to manage blends of innovative and traditional funding sources, whilst finding ways to boost tax revenue for NCDs.

## Background

Non-communicable diseases (NCDs) have come to dominate the global burden of disease but the proportion of all global health financing dedicated to combatting the pandemic has remained constant over the past 15 years at 1–2% [1]. In 2011 ministers responded to this persistent gap at the United Nations (UN) High-Level Meeting on NCDs by committing to ‘explore the provision of adequate, predictable and sustained resources, through domestic, bilateral, regional and multilateral channels, including traditional and voluntary innovative financing mechanisms’ [2]. As the Sustainable Development Agenda stresses domestic responsibility for financing, a surge in overseas NCD spending looks increasingly unlikely [3]. This article presents findings from the expert Working Group convened under the auspices of the World Health Organization (WHO) Global Coordination Mechanism (GCM) on NCDs to examine the current NCD financing landscape and explore sources of funding available to support domestic NCD responses in the Sustainable Development era. The mandate for the report stems from paragraph 45(d) of the Political Declaration of the High-level Meeting of the UN General Assembly on the Prevention and Control of NCDs: ‘to explore the provision of adequate, predictable and sustained resources, through domestic, bilateral, regional and multilateral channels, including traditional and voluntary innovative financing mechanisms’.

The Working Group was chaired by the US health attaché to the UN and the director of health policy research at India’s Institute of Economic Growth. The 12 globally representative members were selected by their home countries and approved by the WHO Director General. The final report was written by Professor Rachel Nugent. This article summarises the findings and situates them in the contemporary financing landscape.

## Non-communicable diseases (NCDs)

Four main conditions account for over 80% of NCD deaths: cancers, respiratory diseases, type II diabetes and cardiovascular disease. These conditions share four common behavioural risk factors: physical inactivity, poor diet, tobacco and excessive alcohol use [4]. In September 2011 ministers acknowledged that NCDs are one of the most pressing development challenges facing mankind [2].

These conditions cause around two thirds of all global deaths and 16 million of these are premature [5]. At the confluence of epidemiological, demographic and economic transitions, low- and middle-income countries (LMICs) experience higher absolute and relative rates of premature death and disability from NCDs [6,7].

The NCD pandemic also levies a heavy toll on international finances. From 2011 to 2025 it is estimated that NCDs will drain over US$ 50 trillion from the global economy [8]. Fortunately, preventing NCDs is very cheap in comparison with the costs of inaction. The WHO’s population-level NCD ‘best buy’ interventions cost US$ < 0.20 per capita per year in low-income countries and US$ < 0.50 in middle-income countries [9]. Scaling up the best buys from 5% to 80% coverage in all LMICs would cost US$ 11.4 billion – less than 5% of current annual health expenditure and almost on par with the leviathan cost of inaction [10,11].

## The contemporary NCD financing landscape

**Figure 1.**
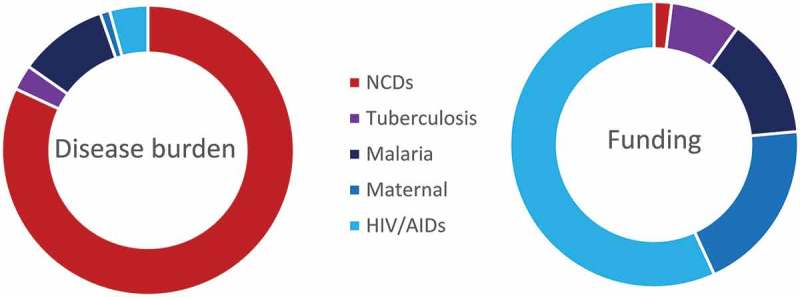
The share of disability adjusted life years (DALYs) and overseas development assistance for health (DAH) for five major disease groups. Data source: Institute for Health Metrics and Evaluation DAH database.

In 2014 NCDs constituted half of the entire global burden of disease, but received less than 2% of all international health aid (US$ 492 million out of US$ 36 billion) [1]. In contrast, HIV represented 4% of the global burden of disease but received 29% of global funds (Figure 1) [5].

Of the $475 million overseas funding devoted to NCDs in 2015, $128 million was provided for mental health and $41 million for tobacco-control [1]. Almost a fifth of NCD funding is from private philanthropy: other major donors include the Gates Foundation and the US and UK governments. These data come from the foremost global health financing accountant; the Seattle-based Institute for Health Metrics and Evaluation (IHME) [12]. The IHME database does not count money that goes towards NCDs that falls under other headings such as ‘health sector support’ and ‘other’, leading to an underestimation of NCD funding. Independent research suggests that overseas NCD financing may be up to 2.5 times higher than currently estimated (although this would still be a mere 3.25% of global health financing) [13,14]. Ministers at the UN General Assembly have asked the Organisation for Economic Co-operation and Development (OECD) Development Assistance Committee to formally track overseas funding to overcome these accounting issues [15].

Data on domestic NCD financing are even scarcer than overseas assistance data. This is despite the fact that domestic financing constitutes 75% of overall health spending in low-income countries and 97% in lower-middle-income countries (where the global burden of NCDs is greatest) [16,17].

In many countries the largest component of domestic health funding comes from over-the-counter payments as patients pay for their own care as and when costs arise. These so called ‘out-of-pocket’ payments contrast with medical insurance (‘pre-pooling’) and can be financially crippling in the case of chronic diseases that require ongoing treatment with expensive medication. Out-of-pocket payments account for 48% of all health expenditure in low-income countries, 36% in middle-income countries and 15% in high-income countries [18]. Despite the fact that NCDs have been the leading cause of mortality in most countries for decades, only half of WHO member states had NCD line items in their health budgets in 2011 [19].

Nugent and Brouwer caution that the NCD-related target (Target 3.4) in the UN Sustainable Development Goals (SDGs) cannot be achieved if funding for NCDs does not increase [20]. Their economic analysis found NCD preventive interventions are expensive to implement but highly cost-effective.

## Funding the post-2015 NCD response

The high-level UN commitment to find new and sustainable sources of NCD financing led to the establishment of the aforementioned expert NCD funding working group, convened under the auspices of the WHO GCM on NCDs. The group identified three areas: domestic, overseas and innovative financing sources.

### Domestic

The working group’s report strongly argues that pre-payment (i.e. insurance) and government provision should replace out-of-pocket expenditure (paying upfront when ill) as the dominant source of NCD funding within LMICs [21]. The chronic nature of NCDs and the high costs of diagnosis and treatment impose heavy financial burdens on impoverished households. Socioeconomic inequalities are exacerbated, families are pushed further into poverty and many simply go without care in settings where out-of-pocket payment is the major source of financing [22–24].

The working group noted that government expenditure is 20.5% of GDP in advanced economies and 16.5% in low-income countries [25]. For governments to assume a greater role in providing NCD prevention and control services – in line with commitments made in 2011, 2014 and 2015 [26–28] – they must raise more revenue for NCDs. The GCM working group identified three ways to meet this aim.

Firstly, governments should seek to allocate a greater share of public funds to NCD control efforts. Signatories of the Abuja Declaration have committed to spending 15% of their annual budget on health and more countries could follow suit in this area [29]. As government revenue grows, additional revenue should preferentially flow to NCDs where these are the leading causes of morbidity and mortality.

Secondly, governments should try to capture the ‘economic dividend’ as continued economic growth generates new funds that can be channelled to fighting NCDs. Over the next two decades the gross domestic product (GDP) of low-income countries is predicted to increase by US$ 1 trillion. Approximately US$ 9 trillion is expected in lower-middle-income countries over the same time period [30]. If tax agencies can collect a share of this dividend, additional resources can be used to increase public spending in all areas.

Thirdly, there is scope for a number of LMICs to raise additional revenue for government spending. There are many ways to boost fiscal capacity including improving tax compliance and the efficiency of revenue collection, increasing tax rates and broadening the tax base, introducing social health insurance contributions for health services, and introducing excise taxes on tobacco, alcohol and unhealthy foods and beverages.

Increased revenue from taxation and long-term economic growth, coupled with commitments to proportionally increase the amount of government expenditure earmarked for NCDs, represents the most sustainable way of financing NCD prevention and control in LMICs.

The working group also encouraged governments to monitor NCD expenditure across all state departments (akin to tracking all sources of overseas funding) and to prioritise poverty reduction strategies that target NCDs. They also encouraged governments to set NCD investment targets and to record this spending in National Health Accounts.

Whilst governments have committed to take the lead in financing national NCD responses, there are many sources of external financing that can make up the shortfall in the short to medium term. These measures are covered in the following sections.

### Overseas development assistance

Donor assistance is an important source of funding, but it can also help to build capacity in LMICs. Shared experts, experience, equipment and approaches can bolster national NCD responses in countries with limited domestic resources [31]. Emerging donors, including BRICS countries (Brazil, Russia, India, China, South Africa) and the Gulf States, are becoming more important in terms of both South–South financial support and in sharing their experience of low-cost approaches to managing NCDs [14,16]. The GCM working group also endorsed the target of spending 0.7% of gross national income on aid. Recipient countries can help by creating clear health performance metrics to offer donors, combined with economic performance measures.

#### Development loans

Development banks are already transitioning from Millennium Development Goals (where NCDs were conspicuously absent) towards the SDGs that include reducing premature NCD mortality by a third [32]. Even in the last year over 20 NCD-inclusive loans worth hundreds of millions of dollars have been supplied by lenders such as the World Bank and the Inter-American Development Bank [33]. Countries have a renewed mandate to apply for international development financing in SDG Target 3.4 and a strong investment case as NCD interventions typically deliver high returns [21].

#### Engaging the private sector

Private capital can be mobilised for NCDs in many ways. Many commercial entities have objectives that closely align with the NCD agenda, such as those operating in the fitness sector, those selling fruits and vegetables, and certain actors in the pharmaceutical industry. Engagement with these businesses can promote the NCD prevention agenda at no cost to the public purse.

Public–private partnerships can raise additional funds for fighting NCDs in countries that are ineligible for traditional donor support. Using public–private partnerships to deliver NCD-related public goods can also increase service quality and release previously untapped sources of private capital [34].

### Innovative financing mechanisms

A broad array of novel financing Initiatives have emerged in recent decades including micro-levies on airplane tickets, credit card rounding plans, and large financing mechanisms like the Global Fund to Fight AIDS, Tuberculosis and Malaria. The UN has made a concerted effort to foster development financing mechanisms that ‘are stable, long-term, that complement official public aid, and that widen the shared benefits of globalisation’ [35,36]. Over US$ 7 billion has been generated for global health issues over the last 15 years using non-traditional financing mechanisms [37]. The innovative financing mechanisms capable of generating additional funds for NCDs broadly fall into the following three categories.

#### Voluntary contributions

Options include credit card plans that round bills up to the nearest dollar and donate the excess monies; lotteries where all profit goes to a specific cause; and the use of targeted marketing schemes to raise additional funds. These innovative ways of raising charitable donations from the general public and corporate sponsors usually earmark funds for a specific use. NCD examples include ‘Go Red for Women’ targeting heart disease and the ‘Pink Ribbon’ campaign for breast cancer.

#### Compulsory levies or taxes

An increasing number of countries are applying ‘sin taxes’ to unhealthy products and using the revenue to promote health. Hypothecated excise taxes on tobacco, alcohol and sugary drinks can all raise money for NCDs whilst promoting prevention [38]. At the international level there is appetite for further hypothecated levies following the UNITAID model of collecting revenue from airline tickets [39]. A mooted Solidarity Tobacco Contribution ‘micro levy’ aims to skim a small percentage from tobacco purchases to raise money for health initiatives [40].

#### Financial mechanisms and facilities

This broad category includes funds from sustainable and impact investing sectors. Microfinance initiatives are an example of a financial mechanism that releases funds to achieve development aims on the local level with private capital from (traditionally) Northern investors. Large financing facilities such as the GAVI Alliance and the Global Fund to Fight AIDS, Tuberculosis and Malaria use a mix of public–private financing and international loans to raise capital.

There is a paucity of innovative financing mechanisms currently focused on NCDs; however, the Global Alliance for Clean Cookstoves Spark Fund is one example [41]. Impact investment in the health sector can benefit NCD responses indirectly by bolstering health systems, primary care and human resources [42]. The market for impact investment in health is large (estimated at US$ 18–123 billion) and is set to expand significantly over coming years [43].

All of the above initiatives can be implemented globally or nationally; however, at every stage there is a risk that contributions will represent reallocations rather than net additions to NCD financing. The working group calls for new additional overseas funds for NCDs rather than reallocation e.g. by redirecting funds from HIV to NCDs. This issue also exists at the national level, especially as most of the working group’s recommendations pertain to raising general government revenue rather than specific NCD funds. Health and finance ministers will have to exercise discipline in ringfencing new funds for NCDs, rather than spending in other sectors.

Directly switching resources from, say, HIV towards NCDs would disrupt established programmes and lead to conflict between health allies. As the global health community shifts emphasis from ‘vertical’ disease-specific funding towards more ‘horizontal’ health system support, NCDs are likely to indirectly benefit. This is because strong health systems are required for prevention, screening, detection, treatment, rehabilitation and palliation. Many of these activities are underdeveloped in LMICs. The establishment of new cadres of health workers, facilities and equipment will benefit patients suffering from all conditions; however, as these services already exist for those with TB but are largely absent for NCDs like hypertension, NCD patients will disproportionately benefit. The same is true for nascent moves to bolster primary care, and from the universal health coverage movement.

A further issue in NCD financing is the sustainability of any new funds. Overseas allocations are based on a set of ever-changing priorities or the whim of private philanthropists. International financing schemes are dependent on market confidence and the performance of the global economy. Government budgets tend to change with every election, and the demographic transition – well underway in countries like China and Bangladesh – will see old-age dependency ratios rise sharply in the coming decades.

## Conclusion

To meet their political commitments to reduce the burden of NCDs, LMICs will have to utilise multiple financing sources. The exact blend of the sources described here will depend on political objectives, fiscal capacity, the domestic burden of disease and the nature of existing relationships with international donors.

The Addis Ababa agreement laid the groundwork for an international agenda that increasingly stresses domestic responsibility for financing. The inward-looking Trump administration in the US has provided additional impetus to strengthen domestic revenue collection. In light of these developments, the first question for many countries is whether domestic financing is a feasible and sustainable option. The next consideration is the amount of external assistance required to catalyse NCD service delivery, followed by which sources of funding are the most appropriate.

LMICs will have to manage an increasingly complex portfolio of funding streams as they transition to financing models that predominantly rely on domestic pre-pooling. Traditional and emerging donors can help to bridge the gap with monetary support and technical assistance. If they are successful the proportion of overseas assistance earmarked for NCDs might justifiably remain low; however, in the short term the disconnect between financing and the global burden of disease is less defensible.

The Sustainable Development Agenda is not an excuse for richer countries to default on commitments to spend 0.7% of gross national income on overseas assistance. Nevertheless, even as resource-poor countries call for more overseas assistance they must begin making provisions for a future characterised by a growing NCD burden coupled with increasing international emphasis on domestic financing. The success of the global NCD response will be largely dependent on the competence of poorly resourced countries in managing increasingly complex blends of funding streams. The future sustainability of NCD funding will be based on domestic and international leaders prioritising NCD prevention and control. LMIC governments will also have to formalise their economies, increase the tax base, and improve the efficiency of their revenue collection agencies to sustain government insurance schemes and safeguard future financing.

## Paper context

Work by the Institute for Health Metrics and Evaluation and others has shown that overseas non-communicable disease (NCD) funding falls well behind other disease groups. This paper describes potential sources of financing for domestic prevention and control initiatives. Countries can use these sources to raise additional monies for NCDs in the future; however, sustained financing depends on the maintenance of NCDs as a global priority.
